# Time course of endothelial damage in septic shock: prediction of outcome

**DOI:** 10.1186/cc3532

**Published:** 2005-05-13

**Authors:** Ortrud Vargas Hein, Klaudia Misterek, Jan-Peer Tessmann, Vera van Dossow, Michael Krimphove, Claudia Spies

**Affiliations:** 1 Department of Anesthesiology and Intensive Care, University Hospital Charité, Campus Mitte, Berlin, Germany

## Abstract

**Introduction:**

Endothelial damage accounts greatly for the high mortality in septic shock. Higher expression of mediators (IL-6, IL-8, soluble intercellular adhesion molecule 1 [sICAM-1], soluble endothelial-linked adhesion molecule 1 [sELAM-1]) have been described for non-survivors in comparison with survivors. We investigated the predictive value of the mediators IL-6, IL-8, sELAM-1 and sICAM-1 and their time course in intensive care unit patients who developed septic shock with respect to outcome.

**Materials and methods:**

We measured serum levels of IL-6, IL-8, sELAM-1 and sICAM-1 in 40 intensive care unit patients who developed septic shock. Measurements were performed until death or until resolution of septic shock. Clinical and laboratory data were also recorded.

**Results:**

After 48 hours the levels of sELAM-1 and sICAM-1 increased in non-survivors and decreased in survivors. sELAM-1 was predictive for outcome on the third day (*P *= 0.02) and the fourth day (*P *= 0.02) after diagnosis of septic shock. This difference in the time course between survivors and non-survivors occurred 7 days before death of the patients (median, 10 days). sICAM-1 levels increased significantly in non-survivors over the study period (*P *< 0.001). sELAM-1 (*P *= 0.04), IL-6 (*P *= 0.04) and IL-8 (*P *= 0.008) were significantly higher in non-survivors over the whole study period. The age and norepinephrine dose >0.5 μg/kg/min were significantly different between the groups.

**Conclusion:**

sELAM-1 showed a markedly opposing course after 48 hours of septic shock. This adhesion molecule may be a useful early predictor of disease severity in the course of septic shock after early initial treatment of the patients, and might suggest considering endothelial-restoring therapy.

## Introduction

Endothelial damage accounts for much of the pathology of sepsis, resulting in capillary leak, hypotension, microvascular thrombosis with consecutive tissue hypoxia and, finally, multiple organ failure (MOF) and lethal outcome [1-3]. Endothelial damage is worsened in septic shock [4]. The mortality of septic shock is higher than the mortality in sepsis (35–60% versus 20–40%) [4,5]. The release of cytokines (IL-6, IL-8) and adhesion molecules (soluble endothelial-linked adhesion molecule 1 [sELAM-1], soluble intercellular adhesion molecule 1 [sICAM-1]) has been shown to correlate well with endothelial damage in an experimental setting – especially for sELAM-I, which is specific for endothelial tissue [2,6,7]. Although the release of these mediators is not only sepsis related, the levels are significantly higher in sepsis and in septic shock than after trauma, postoperatively or after myocardial infarction [8-12]. In addition, these mediators have higher levels in non-survivors than in survivors, and the baseline levels have been correlated with outcome [2,3,8,10-15].

The time of admission to the study and the onset of therapy are of major relevance for outcome, however, as shown by Rivers and colleagues in the early goal-directed therapy study in severe sepsis and septic shock patients [16]. As early clinical intervention improves outcome and as there are increasing levels of cytokines in non-survivors, in comparison with a decrease in survivors, differences in the mediator time course between survivors and non-survivors after early onset of therapy could be predictive for the outcome and for trend-setting for further therapy measures [10,11,15,17-19].

We investigated the predictive value of the mediators IL-6, IL-8, sELAM-1 and sICAM-1 and their time course, as primary outcome measures, in intensive care unit (ICU) patients who developed septic shock with respect to outcome. In addition, IL-8 as an early chemoattractant cytokine and IL-6 as an inflammatory tissue damage marker were investigated. Clinical data, such as age, the use of hemodynamically active substances and myocardial ischemia, were investigated as secondary outcome measures.

## Materials and methods

### Patients

After ethical committee approval and written informed consent from the legal representatives, 42 patients suffering from septic shock were enrolled in this observational study. Two patients had to be excluded after enrollment because of imminent surgery, so 40 patients completed the study. All patients fulfilled the clinical and laboratory criteria of septic shock as outlined in the 1992 Consensus Conference [20]. Exclusion criteria were age <18 years, pregnancy, patients who have had surgery within 48 hours before inclusion and patients who have had cardiac surgery and neurosurgery. Patients with an acute history of severe cardiac insufficiency (New York Heart Association class III-IV) [21] and coronary artery disease before the development of septic shock were also excluded [22].

### Monitoring and management

The study was initiated in the first 24 hours after septic shock had been diagnosed. All patients were already admitted to the ICU and were under ICU standard therapy and monitoring [23]. All patients received analgesia, sedation and mechanical ventilation. The patients were screened twice a day. The study ended in the case of death or in resolution of septic shock.

A fiber optic pulmonary artery flotation catheter (Baxter Swan-Ganz^® ^Intelicath™ continuous cardiac output thermodilution catheter 139H, 7.5 Fr; Baxter/Edwards Critical-Care, Irvine, CA, USA) and a radial artery catheter were inserted as part of the routine for continuous cardiovascular monitoring in septic shock. Hemodynamic measurements were recorded at study entry and every 8 hours during the study. Fluids were given to achieve an optimal left atrial pressure. After adequate fluid resuscitation, norepinephrine (maximum 4.0 μg/kg/min) was titrated to maintain a mean arterial pressure >70 mmHg. Catecholamine therapy in the case of low-output failure was performed primarily with dobutamine (maximum 20 μg/kg/min) or dopamine (maximum 10 μg/kg/min) at the discretion of the physician on duty. Enoximone (maximum 10 μg/kg/min) was added if low-output failure persisted, and then epinephrine infusion (maximum 2.0 μg/kg/min) was initiated if low-output failure remained. The target value was a cardiac index >3.0 l/min/m^2^. The amount of different positive inotropic substances was expressed as the number used in each group.

A 12-lead Holter electrocardiogram (ECG) was recorded every 8 hours to determine possible myocardial ischemia, defined by Spies and colleagues [22]. The oxygenation index was calculated as the quotient of partial arterial oxygen pressure and the inspired oxygen fraction (mmHg).

### Group assignment

It was decided *a priori *to assign patients to the survivors group when they were discharged from the ICU to a regular ward. Those patients who died due to septic shock were assigned to the non-survivors group. Patients who died from a cause other than septic shock and consecutive MOF during their ICU stay were excluded from the study.

### Laboratory data

Blood gas analysis was performed every 8 hours to determine the levels of hematrocrit and hemoglobin, and the arterial partial oxygen pressure (ABL 500; Radiometer, Copenhagen, Denmark).

Creatin kinase and the creatin kinase-myocardial bands were determined every 8 hours (BM/Hitachi 717 analyser; Boehringer Mannhein, Inc., Mannheim, Germany). The creatin kinase/creatin kinase-myocardial band fraction was calculated and a result >6% was recorded positive for myocardial ischemia [22]. Blood samples for the determination of IL-6 concentrations (Enzymeimmunoassay [Milenia^®^]; DPC Biermann GmbH, Bad Nauheim, Germany), of IL-8 concentrations (Enzymeimmunoassay [Milenia^®^]; DPC Biermann GmbH), of sICAM-1 concentrations (enzyme immunoassay kit BBE 1b; R&D Systems, Minneapolis, MN, USA), of sELAM-1 concentrations (enzyme immunoassay kit BBE 2b; R&D Systems) and of troponin T concentrations (enzyme-linked immunosorbent assay Enzymun-Test™ batch ELISA ES 300 analyser; Boehringer Mannheim Inc.) were withdrawn every 8 hours and were centrifuged, and the plasma was stored at -80°C until analysis.

### Statistical analysis

Data are expressed as the median and range. Intergroup statistical analysis for determined time intervals was performed using the Mann–Whitney U test for continuous variables and using the Pearson chi-square test for dichotomous variables. Intragroup statistical analysis for the determined time intervals was performed with the Wilcoxon matched-pairs signed-rank sum test. For intergroup and intragroup analysis over the whole study period, the two-factorial non-parametric (analysis of variance)-type rank variance analysis for longitudinal data and small sample sizes using the SAS System software (SAS Institute Inc., Cary, NC, USA) was used. Variables that were significantly different between groups were analysed as predictors for outcome (group variable, survivor/non-survivor), determining the area under the receiver operating characteristics curve (AUC). The AUC, the *P *value and the 95% confidence intervals are stated. *P *< 0.05 was considered statistically significant.

## Results

Forty patients were included in the study and 16 (40%) patients were discharged from the ICU to a normal ward. Twenty-four (60%) patients died due to septic shock. Patients in the non-survivor group were significantly older and stayed a significantly shorter time in the ICU than the survivors (Table 1). Survivors had a significantly higher rate of pneumonia as the sepsis focus whereas non-survivors had a significantly higher rate of peritonitis as the focus (Table 1). The Acute Physiology and Chronic Health Evaluation III baseline score and the Acute Physiology and Chronic Health Evaluation III maximum score did not significantly differ between the groups (Table 1). All patients required norepinephrine therapy but significantly more non-survivors than survivors required norephinephrine infusion >0.5 μg/kg/min (Table 2). The number of positive inotropic agents necessary and the markers for myocardial ischemia (monitored by ECG), for creatin kinase/creatin kinase-myocardial band fraction >6% and for troponin T were not significantly different between survivors and non-survivors (Table 2).

**Figure 1:**
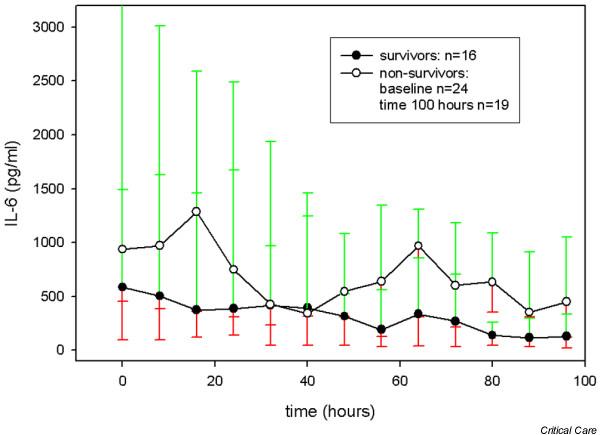
IL-6 for survivors and non-survivors over time.

**Figure 2:**
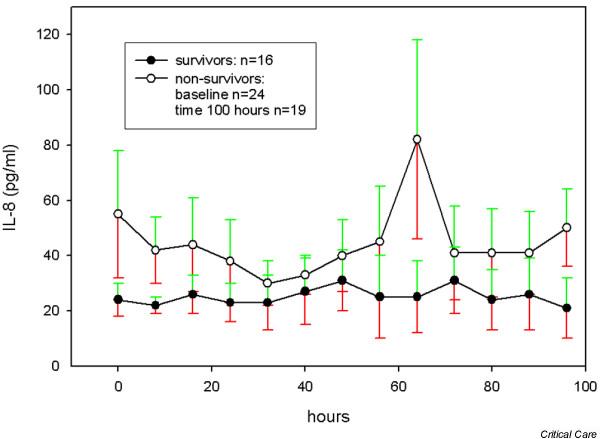
IL-8 for survivors and non-survivors over time.

Intergroup analysis of variance between survivors and non-survivors showed significantly higher levels for IL-6 (*P *= 0.04), for IL-8 (*P *= 0.008) and for sELAM-1 (*P *= 0.04) in the non-survivors group. sICAM-1 (*P *= 0.25) was not significantly higher in levels in the non-survivors group. The intragroup analysis for IL-6 showed a significant decline between the first value and the last value (before discharge from the study or death) for survivors (*P *= 0.002) and non-survivors (*P *= 0.04) (Fig. 1). The intragroup analysis for IL-8 between the first value and the last value (before discharge from the study or death) was not significantly different in both groups (survivors, *P *= 0.17; non-survivors, *P *= 0.78) (Fig. 2).

**Figure 3:**
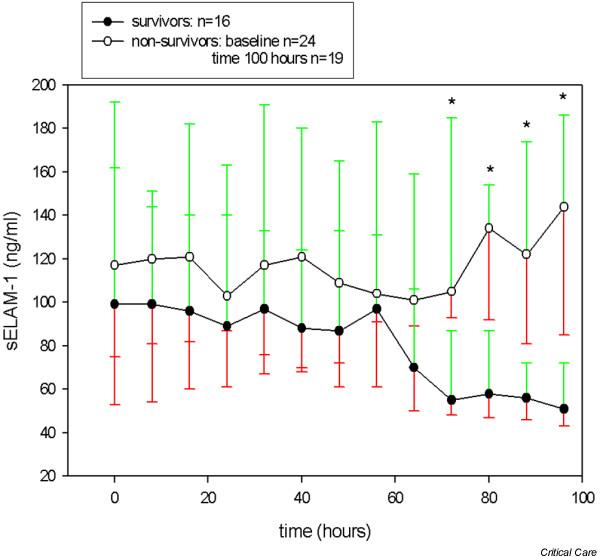
Soluble endothelial-linked adhesion molecule 1 (sELAM-1) for survivors and non-survivors over time. * Significant difference (*P *< 0.05) for sELAM-1 between survivors and non-survivors.

**Figure 4:**
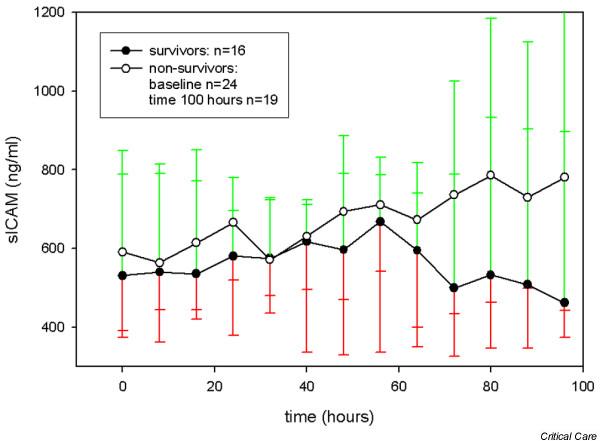
Soluble intercellular adhesion molecule 1 (sICAM-1) for survivors and non-survivors over time.

After a comparable course in the first 2 days, non-survivors showed an increase in median values of sELAM-1 and sICAM-1 whereas survivors' adhesion molecule levels decreased markedly (Figs 3 and 4). This increase was significant for sICAM-1 in the non-survivor group when comparing the first value with last value before discharge from the study or death of the patients (*P *< 0.001) (Fig. 4). The marked decline of median values for sELAM-1 in the survivor group was significant in the comparison of the first time point with the last time point before discharge from the study or death (*P *= 0.04) (Fig. 3). When comparing survivors and non-survivors at single time points, sELAM-1 was significantly higher in non-survivors from the third day onwards (*P *= 0.02) (Fig. 3).

The AUC values for baseline, the third day and the fourth day measurements of IL-6, IL-8, sELAM-1 and sICAM-1 are presented in Table 3. IL-8 was most predictive for outcome at baseline, and sELAM-1 most predictive on the third and fourth days (Table 3). The AUC for age (AUC, 0.761; *P *= 0.01; 95% confidence interval, 0.624–0.898) and that for median norepinephrine dosage (AUC, 0.766; *P *= 0.001; 95% confidence interval, 0.636–0.896) were also significantly predictive for outcome.

## Discussion

The most important finding in this study was the different time courses of the markers of endothelial damage (sELAM-1 and sICAM-1) after the second day in survivors and non-survivors of septic shock. After a comparable course at different levels in the first 2 days, non-survivors had an increase in adhesion molecule concentrations whereas survivors' adhesion molecule levels decreased markedly. SELAM-1 was predictive for outcome on the third and fourth days after the diagnosis of septic shock. This difference in time courses between survivors and non-survivors was evident on the third day and, therefore, far before death of the patients (median, 10 days).

Endothelial damage accounts for much of the pathology of septic shock, resulting finally in MOF and lethal outcome [1-3]. sELAM-1 is specific for endothelial tissue [2,7]. The latter marker and sICAM-1 have been shown to be significantly elevated at baseline and inconsistent in levels over the whole study period in sepsis, in comparison with trauma patients or critically ill patients without sepsis [2,3,8-12]. The levels of adhesion molecules in septic shock patients have been described as markedly elevated at baseline in comparison with septic patients without shock [10,12,24]. In addition, sELAM-1 and sICAM-1 have been shown to be markedly elevated at baseline in non-survivors in comparison with survivors, as shown in the present study [2,8,10-12,24].

In the present study, non-survivors (in comparison with survivors) showed elevated adhesion molecule levels over the whole study period. After a comparable time course at different levels over the first 48 hours, the endothelial mediator levels increased in non-survivors and decreased in survivors. sELAM-1 was predictive for outcome at the third and fourth days. Kayal and colleagues investigated patients with severe sepsis (56%) and with septic shock (44%) on admission to the ICU or during ICU hospitalisation. Seventy-two percent of the septic shock patients had a putative sepsis onset >6 hours before inclusion in the study, and 82% of the septic shock patients died after a median time of 3 days [10]. Fifty percent of the severe sepsis patients had a putative sepsis onset >6 hours before inclusion into the study, 14% of which died after 6 days in the ICU [10]. Kayal and colleagues observed an increase in sICAM-1 and sELAM-1 levels for 3–4 days after study inclusion in non-survivors, sELAM-1 then returning to levels similar to those observed in survivors whereas sICAM-1 continued to increase in non-survivors [10]. Those authors concluded that baseline sICAM-1 and sELAM-1, as markers of endothelial cell activation, predicted disease severity – and sICAM-1 more then sELAM-1 reflected the intensity of inflammation and tissue damage in late sepsis [10].

Boldt and colleagues investigated septic patients already admitted to the ICU at the onset of sepsis, 40% of which died [11]. The authors also demonstrated that sELAM-1 decreased over time in septic patients while sICAM-1 increased further [11]. Cowley and colleagues investigated adhesion molecule levels of patients admitted to the ward or the ICU within 12 hours after the onset of systemic inflammatory response syndrome, with or without signs of organ dysfunction or hypoperfusion – 60% of them died [18]. This study group observed increased levels of sELAM-1 over the study period in patients with sustained organ dysfunction and in non-survivors, whereas sELAM-1 levels decreased in patients whose organ dysfunction resolved [18]. Sessler and colleagues measured sICAM-1 levels of septic patients (64% in septic shock, from which 75% died) within 12 hours after admission to the ICU for sepsis, of which 48% died [12]. The authors were able to show that baseline sICAM-1 levels correlate independently with outcome [12]. Cummings and colleagues investigated sELAM-1 levels within 24 hours of admission to the ICU of 119 critically ill patients (7% had no systemic inflammatory response syndrome, 37% had non-infectious systemic inflammatory response syndrome, 56% were septic, 34% were in shock) [24]. The authors found a modest correlation between day 1 sELAM-1 levels and organ dysfunction as well as survival [24].

The inclusion time of patients into the study could be crucial for the course and interpretation of mediator levels in relation to outcome [17]. If admission and therapy is delayed, mediator levels might already be high at admission [17]. The clinical signs of septic shock become evident when the inflammatory insult is already ongoing and initialising therapy might be delayed, leading to a worse outcome [16]. The early goal-directed therapy performed by Rivers and colleagues in septic shock patients provided a significant outcome benefit [16]. Our patients, who were already under standardised ICU therapy before septic shock began, died 7 days (median) after possible outcome prediction by enhanced endothelial damage markers in non-survivors. The monitoring of sELAM-1 and sICAM-1 over the time course of septic shock could probably indicate when the patients' course is leading to lethal outcome and could help physicians to intervene and monitor further therapy before the patients die. Such therapies aim at recruiting the endothelium; for example, the application of activated protein C.

IL-6 has been described to have pro-inflammatory and anti-inflammatory properties in different animal and human septic and non-septic models [2,15,25,26]. IL-6 is widely accepted as a marker for disease severity in septic shock but elevations are not sepsis specific [13,15,27-29]. However, as has been demonstrated for adhesion molecules, IL-6 levels in septic shock patients were significantly higher and stayed higher in non-survivors than in survivors, as shown in the present study [13-15,17,27,28]. The predictive value of IL-6 on admission has been described for septic patients and septic shock patients [14,15,19]. Baseline values in our study were not predictive for outcome, perhaps because of the early entry time into the study as described earlier.

IL-6 tended to correlate with outcome on the third and fourth days after onset of septic shock. Pinsky and colleagues described the persistence of high levels of IL-6, and not the peaks of IL-6, as being predictive for outcome [17]. IL-6 continuously dropped in survivors whereas it showed a variable course in non-survivors. This variability has been described in patients suffering from sepsis and from septic shock [13,15]. In both groups, however, IL-6 levels decreased significantly from admission until the end of this study, in contrast to other cytokines such as tumor necrosis factor alpha or to other adhesion molecules, as shown in other studies and our own [14,15]. Presterl and colleagues observed a steady decrease in IL-6 over a 7-day period in survivors and observed persistent high levels in non-survivors [13]. This course could be related to an initial pro-inflammatory characteristic and a later anti-inflammatory characteristic of IL-6 when compared with the explicit pro-inflammatory cytokine tumor necrosis factor alpha [14,15,25,26].

IL-8 was significantly higher in non-survivors than in survivors, and it was predictive for lethal outcome at baseline. IL-8, a chemoattractant, is an early pro-inflammatory component released in sepsis by endothelial cells and other cells [7]. High levels of IL-8 have been described in sepsis, in shock and in MOF with poor outcome, consistent with our study [29-31]. These results, however, are conflicting in the literature [29,31,32]. Especially for early detection of nosocomial pneumonias and newborn infections, IL-8 has been shown to be an adequate marker and predictor [33-36]. The predictive value of this parameter at baseline, as shown in the present study, might be a hint that patients were in the phase of early septic progression.

The rate of pneumonias and peritonitis as the septic focus was significantly different between survivors and non-survivors. After revision of the literature, no data could be found regarding possible differences in expression of endothelial damage markers and outcome looking at different infection sites.

All our patients required norepinephrine therapy. Significantly more non-survivors needed norepinephrine at a dose >0.5 μg/kg/min than survivors, probably due to profound volume-refractory vasodilation. Norepinephrine follows dopamine as the first-choice vasopressor in septic shock and has been applied in dosages as high as 5 μg/kg/min [4,37]. The use of positive inotropic therapy to achieve supramaximal hemodynamic values for oxygen delivery, for mixed venous oxygen saturation and for cardiac index has been reported to worsen the outcome of patients in septic shock [38-40]. In the present study the use of positive inotropic therapy did not differ between survivors and non-survivors. Although myocardial dysfunction has been extensively described in sepsis, the main pathophysiology developing in septic shock is the peripheral vasodilation with consecutive hypotension [4,37,41,42]. As myocardial dysfunction/ischemia may be contributing factors influencing study results and the outcome, patients with an acute history of severe cardiac insufficiency and coronary artery disease before the development of septic shock were excluded from the study. The laboratory parameters for myocardial ischemia and the ECGs performed did not show differences in signs of myocardial ischemia between survivors and non-survivors. The high incidence of myocardial ischemic signs observed in the ECGs has to be interpreted carefully. Other studies have described the low specificity of ECG in comparison with troponin T for the diagnosis of myocardial ischemia [8].

Patients in the non-survivor group in this study were significantly older than the survivors. Age was also a significant predictor of lethal outcome in the AUC analysis. The patients' age has been described as a risk factor of fatal outcome in patients with sepsis, explained by a possibly diminished physiologic reserve and a poor immune status [1,5,19,43]. Boldt and colleagues were able to show higher levels of sELAM-1 and sICAM-1 in patients older than 70 years in comparison with patients younger than 50 years, indicating an association with more extensive endothelial damage [43]. In the present study, sELAM-1 was significantly higher in patients older than 65 years (*P *= 0.01). When excluding non-survivors, however, sELAM-1 was no longer significantly higher in patients older than 65 years (*P *= 0.60).

A major limitation of the present study is the low number of patients. This fact could be the cause for the large range in standard deviation of the markers measured. A far greater number of patients will be needed to verify the results presented.

## Conclusion

The endothelial marker sELAM-I showed a markedly opposing and predictive course after 48 hours of septic shock. Our data suggest that the adhesion molecule sELAM-1 might be useful in assessing disease severity in the course of septic shock after early initiation of treatment. This might provide a valuable means of monitoring and a means of guidance of therapy with substances known to reduce endothelial damage (such as, for example, activated protein C).

## Key messages

• 	sELAM-1 showed early prediction of outcome in septic shock patients

## Abbreviations

AUC = area under the receiver operating characteristics curve; ECG = electrocardiogram; ICU = intensive care unit; IL = interleukin; MOF = multiple organ failure; sELAM-1 = soluble endothelial-linked adhesion molecule 1; sICAM-1 = soluble intercellular adhesion molecule 1.

## Competing interests

The author(s) declare that they have no competing interests.

## Authors' contributions

OVH and CS completed the proposal writing and experimental design. OVH, J-PT and KM participated in the research coordination, data analysis, presentation and conduction of all experimental aspects of the study. OVH, VvD, MK and CS prepared the manuscript.

## Figures and Tables

**Table 1:** Baseline and outcome data

	Survivors (*n *= 16, 40%)	Non-survivors (*n *= 24, 60%)	P*
Age (years)	59 (28–82)	65 (33–86)	0.03
Sex (male/female)	11/5	11/13	0.15
Intensive care unit stay (days)	27 (11–48)	8 (2–57)	<0.01
Sepsis focus (*n*)			
Pneumonia	9	4	0.02
Peritonitis	2	13	0.04
Wound infection	3	4	>0.99
Abscess	2	3	>0.99
Hemoglobin (g/dl)	10 (7–14)	11 (7–13)	0.61
Oxygenation index (mmHg)	245 (114–421)	199 (89–384)	0.44
APACHE III baseline score	55 (23–88)	61 (12–100)	0.91
APACHE III maximum score	75 (52–108)	86 (52–117)	0.47
MODS baseline	6 (2–11)	7 (2–12)	0.36
MODS max	9 (5–15)	9 (4–14)	0.73

Data presented as median (range). APACHE, Acute Physiology and Chronic Health Evaluation; MODS, multiple organ dysfunction syndrome.

*P value for intergroup baseline and outcome data: Mann–Whitney U test, and Pearson chi-square and Fisher exact tests, respectively.

**Table 2:** Clinical and laboratory data

	Survivors (*n *= 16, 40%)	Non-survivors (*n *= 24, 60%)	P*
Norepinephrine (*n*)	16 (100%)	24 (100%)	
Norepinephrine >0.5 μg/kg/min mean values (*n*)	8	22	<0.01
Number of + inotropic medications (dobutamine or dopamine, enoximone and epinephrine) (*n*)			0.79
0	3	6	
1	9	11	
2	4	6	
3	0	1	
Myocardial ischemia signs in electrocardiogram (*n*)	12	15	0.41
Troponin T >0.2 (ng/ml)	5	7	0.89
CK/CK-MB fraction >6%	1	0	0.22

Data presented as median (range). CK/CK-MB, creatin kinase/creatin kinase-myoglobin band.

*P value for intergroup data analysis: Pearson chi-square and Fisher exact tests.

**Table 3:** Predictive parameters determined by the area under the receiver operating characteristics curve (AUC)

Time point	AUC	95% confidence interval	*P*
Baseline			
IL-8	0.777	0.619–0.935	0.004
IL-6	0.648	0.462–0.834	0.14
sELAM-1	0.600	0.400–0.800	0.30
sICAM-1	0.548	0.360–0.735	0.622
Third day			
IL-8	0.133	0.402–0.923	0.25
IL-6	0.727	0.501–0.953	0.087
sELAM-1	0.808	0.599–1.017	0.02
sICAM-1	0.677	0.427–0.927	0.18
Fourth day			
IL-8	0.775	0.529–1.021	0.05
IL-6	0.737	0.504–0.971	0.09
sELAM-1	0.847	0.631–1.064	0.02
sICAM-1	0.694	0.433–0.956	0.18

sELAM-1, soluble endothelial-linked adhesion molecule 1; sICAM-1, soluble intercellular adhesion molecule 1.
